# Design Features for Linguistically-Mediated Meaning Construction: The Relative Roles of the Linguistic and Conceptual Systems in Subserving the Ideational Function of Language

**DOI:** 10.3389/fpsyg.2016.00156

**Published:** 2016-02-19

**Authors:** Vyvyan Evans

**Affiliations:** Bangor UniversityBangor, UK

**Keywords:** meaning construction, language faculty, access semantics, LCCM theory, design features for meaning

## Abstract

Recent research in language and cognitive science proposes that the linguistic system evolved to provide an “executive” control system on the evolutionarily more ancient conceptual system (e.g., Barsalou et al., 2008; Evans, 2009, 2015a,b; Bergen, 2012). In short, the claim is that embodied representations in the linguistic system interface with non-linguistic representations in the conceptual system, facilitating rich meanings, or *simulations*, enabling linguistically mediated communication. In this paper I build on these proposals by examining the nature of what I identify as *design features* for this control system. In particular, I address how the *ideational function* of language—our ability to deploy linguistic symbols to convey meanings of great complexity—is facilitated. The central proposal of this paper is as follows. The linguistic system of any given language user, of any given linguistic system—spoken or signed—facilitates access to knowledge representation—concepts—in the conceptual system, which subserves this ideational function. In the most general terms, the human meaning-making capacity is underpinned by two distinct, although tightly coupled *representational systems*: the conceptual system and the linguistic system. Each system contributes to meaning construction in qualitatively distinct ways. This leads to the first design feature: given that the two systems are representational—they are populated by *semantic representations*—the nature and function of the representations are qualitatively different. This proposed design feature I term *the bifurcation in semantic representation*. After all, it stands to reason that if a linguistic system has a different function, vis-à-vis the conceptual system, which is of far greater evolutionary antiquity, then the semantic representations will be complementary, and as such, qualitatively different, reflecting the functional distinctions of the two systems, in collectively giving rise to meaning. I consider the nature of these qualitatively distinct representations. And second, language itself is adapted to the conceptual system—the semantic potential—that it marshals in the meaning construction process. Hence, a linguistic system itself exhibits a bifurcation, in terms of the symbolic resources at its disposal. This design feature I dub *the birfucation in linguistic organization*. As I shall argue, this relates to two distinct reference strategies available for symbolic encoding in language: what I dub *words-to-world reference* and *words-to-words reference*. In slightly different terms, this design feature of language amounts to a distinction between a *lexical subsystem*, and a *grammatical subsystem*.

## Introduction

This paper relates to broad research programme concerning the nature of the human capacity for language, and investigating what makes language special. For much of the second half of the twentieth century investigating this capacity has often been driven, in general terms, by asking the following question: What is the nature of language? One response to this question, and the prevailing view in Anglo–American language science, at least until relatively recently, was that it would ultimately be possible to identify principles, specific to language, that accounted for what makes it unique (e.g., Hauser et al., 2002). In particular, such principles were presumed to make language functionally distinct from other aspects of human cognition, as well as qualitatively distinct from, and functionally far more sophisticated than the communicative systems exhibited by other species. And these principles were assumed to be part of the human genetic-endowment. This functional specificity—a species-specific feature of the human mind—is often referred to as the *language faculty*, and is embodied most notably in the tradition pioneered by Chomsky (1965), and thereafter.

In this paper, I propose a somewhat different perspective. And this arises as I begin, by asking a slightly different question. My starting point is to ask: what is language for? It is presumably unarguable that, from the perspective of language as a communicative system, it exhibits two main functions. The first can be characterized as an ideational function: language serves to convey ideas, ranging from stating one’s name, making an idle comment on the weather, to declaring undying love (e.g., Evans, 2009, 2015b). And the second can be characterized as an interactive-interpersonal function (e.g., Levinson, 2006; Heine and Kuteva, 2007; Tomasello, 2008). Here language serves to signal intentional actions: actions in the sense that linguistic utterances have illocutionary force (Searle, 1969)—they attempt to influence the mental states, wishes, feelings and behavior of our interlocutor, influencing and even changing aspects of the world in the process.

Evidence for the interpersonal-interactive function of language comes from grammatical organization, as well as language-specific discourse conventions, demonstrating that language is fundamentally dyadic in nature. For instance, the languages of the world virtually all appear to include a pronoun system that maintains a role for second-person (‘you’). Seemingly universal aspects of linguistic organization such as interrogatives (questions), imperatives (commands), and deontic modality (e.g., *You may*…), provide linguistic resources that seek to influence others (Heine and Kuteva, 2007). Moreover, knowledge of language use includes a complex system of turn-taking conventions associated with competent language use during ongoing discourse (Sacks et al., 1974; Sacks, 1992). Finally, languages appear to universally assume a speaker-hearer distinction. The distinction in English, for instance, between definite vs. indefinite articles (e.g., *the* vs. *a*), is evidence of this: the use of the indefinite article signals that while the entity being referred to is known by the speaker, it is left as unidentified for the hearer. Similarly, spatial deictic expressions—terms that take their reference from the speaker’s spatial location, ‘deictic’ derives from the Greek *deixis*, meaning ‘pointing’—in languages often assume a speaker-hearer dichotomy.

An example of spatial deixis, in English, isthe deictic expression *this*, which designates something proximal to speaker (and hearer), while *that* points to an entity that is distal. Some language have spatial deictics that disambiguate between proximity and distance from speaker and hearer, for instance ‘close to speaker, but not hearer,’ and ‘distal to speaker, but not hearer.’ Lexical items such as these again point to an organizational principle involving a speaker/hearer, and hence an interpersonal-interactive context.

For language to facilitate its interactive-interpersonal function, it stands to reason that language must have evolved as a means of expressing ideational complexity (Hurford, 2007). After all, we can, presumably, only influence the mental states of others once we have a fairly sophisticated symbolic means of expressing our own thoughts and feelings, in a bid to encode and externalize these, in order to have an impact on others and the world around us. And while the two communicative functions may have co-evolved—the interpersonal-interactive function may have led to increased ideational complexity, a more sophisticated means of expressing ideational complexity, in turn, enhanced our ability to engage interactively with others (e.g., Deacon, 1997; Hurford, 2012; Evans, 2015b)—in this paper, I primarily focus on the ideational function of language.

In particular, I argue that language, in fulfilling its ideational function, takes advantage of the *semantic potential* of the evolutionarily prior conceptual system, a system that, in outline at least, we share with other great apes—gorillas, chimpanzees, bonobos, and orangutans—primates more generally, and indeed, many other mammalian species (e.g., Barsalou, 2005; Call and Tomasello, 2008; Hurford, 2012). From this perspective, what makes language special is not that it is functionally distinct, for instance, an informationally encapsulated faculty or module of mind (e.g., Fodor, 1983). On the contrary, the linguistic system of any given language user, of any given linguistic system—spoken or signed—facilitates access to knowledge representation—concepts—in the conceptual system, in order to construct meaning, during the course of communication. The relationship, then, between a linguistic system, and the human conceptual system, is that of a symbiotic assembly, co-evolved and co-adapted in order to enable meaning construction in the course of communication (Evans, 2015a,b). In order to achieve this, this meaning-making complex exhibits a number of design features, facilitating meaning construction. In this article I examine the two central design features of this meaning-making complex, which enable human meaning construction: the design features for a bifurcation in semantic representation, and for a bifurcation in linguistic organization.

In the most general terms, the human meaning-making capacity is underpinned by two distinct, although tightly coupled *representational systems*: the conceptual system and the linguistic system. Each system contributes to meaning construction in qualitatively distinct ways. This leads to the first design feature: given that the two systems are representational—they are populated by *semantic representations*—the nature and function of the representations are qualitatively different (see The Bifurcation in Semantic Representation Design Feature). After all, it stands to reason that if a linguistic system has a different function, vis-à-vis the conceptual system, which is of far greater evolutionary antiquity, then the semantic representations will be complementary, and as such, qualitatively different, reflecting the functional distinctions of the two systems, in collectively giving rise to meaning. I consider the nature of these qualitatively distinct representations.

Second, language itself is adapted to the conceptual system—the semantic potential—that it marshals in the meaning construction process (see The Bifurcation in Linguistic Organization Design Feature). Hence, a linguistic system itself exhibits a bifurcation, in terms of the symbolic resources at its disposal. As I shall argue, this relates to two distinct reference strategies available for symbolic encoding in language: what I dub *words-to-world reference* and *words-to-words reference*. In slightly different terms, this design feature of language amounts to a distinction between a *lexical subsystem*, and a *grammatical subsystem*.

## Background

In this section I present the proposal that the conceptual and linguistic systems have distinct, albeit complementary, functions in subserving the ideational function of language. This section provides the necessary background for discussion of the two design features, that enable this, later in the paper.

### The Conceptual and Linguistic Systems

In previous work (Evans, 2009, 2015b), I have argued that human-like meaning-making is contingent upon a bifurcation in the two representational systems upon which linguistically mediated communication depends. Linguistic communication is contingent on an evolutionarily prior conceptual system. The human conceptual system, shared, at least in outline with the other great apes, evolved not for communication, but rather for functions such as reason, choice, learning, categorization and advance planning, in the quotidian world of threat and opportunity (Evans, 2009). Much later, and probably for much of the 2.8 million years of the evolutionary trajectory of the genus *Homo*, a linguistic system has been evolving—built on the *cooperative intelligence* that emerged with the genus *Homo* (Evans, 2015b; see Deacon, 1997; Tomasello, 2014). And the linguistic system makes use of the qualitatively distinct representational format of the conceptual system, for purposes of communication. On this account, language provides a means of bootstrapping representations in the conceptual system for linguistically mediated communication (Evans, 2015a,b).

Our species shares in outline, especially with the other great apes, a complex conceptual system (e.g., Barsalou, 2005; Hurford, 2007, 2012; Evans, 2009). A conceptual system evolved not for communication, but for a range of more pressing, quotidian concerns, such as categorization, learning, forward-planning, way finding, and so on (Barsalou, 1992). But while many higher-order species possess sophisticated conceptual systems, humans appear to be alone in possessing language (e.g., Evans, 2014). In addition, the conceptual prowess of humans, as manifested, perhaps most notably, by the ideational and material culture characteristics of all human groups, is both quantitatively and qualitatively distinct from any other extant species (Tomasello, 1999, 2014).

One implication of this fact is that it may be language—and the cognitive and biological changes that were necessitated by it over the 2.8 million years of the ancestral human evolutionary trajectory—that has provided the sine qua non: language may be the key in unlocking the otherwise mute semantic potential of the human conceptual system (see, for instance, Mithen, 1996; Deacon, 1997; Evans, 2015b).

From this perspective, a linguistic system provides our species with added value: it provides an “executive” control function—an idea I shall develop during the course of the paper, operating over embodied concepts in the conceptual system (Barsalou, 2005; Barsalou et al., 2008; see also Evans, 2009). The idea I advance here is that language provides the framework that facilitates the composition of concepts for purposes of communication. This is achieved as language consists of a grammatical system, with words and grammatical constructions cueing activations of specific body-based states in the brain (Bergen, 2012: Chapter 5). On this account, language allows us to control and manipulate the conceptual system, which, after all, must have originally evolved for more rudimentary functions, such as object recognition and classification. Under the control of language, we can make use of body-based (not exclusively sensorimotor) concepts in order to develop abstract thought.

In short, representations in the linguistic system co-conspire with representations in the conceptual system in the process of meaning construction. And accordingly, the linguistic representations must have evolved to complement concepts in the conceptual system; accordingly, it stands to reason that the nature of linguistic representations must have a different quality from the rich, multimodal concepts in the conceptual system. After all, if language really does provide an executive control function, specialized for tapping into the conceptual system’s meaning potential, then it stands to reason that language evolved a complementary function; the nature of semantic representation in language must be qualitatively different from the representations—concepts—that populate the conceptual system.

### Evidence for the Embodied Nature of Concepts

Before continuing, I briefly review some of the evidence for thinking that the conceptual system is populated by representations that are embodied in nature. The embodied (or grounded) embodied cognition account of concepts blurs the distinction between perception/interoception and cognition (e.g., Barsalou, 1999, for an early, influential account). On this view, concepts are directly grounded in the perceptual and interoceptive brain states that give rise to them. This embodied cognition perspective takes a modal view of concepts: the semantic substrate of concepts is directly grounded in, and arises from, the sorts of modalities that the concept is a representation of (see Barsalou, 2008 and Shapiro, 2010 for reviews. Notable exemplars of this view include e.g., Damasio, 1994; Clark, 1997; Glenberg, 1997; Barsalou, 1999; Lakoff and Johnson, 1999; Zwaan, 2004; Gallese and Lakoff, 2005; Chemero, 2009; Evans, 2009; Vigliocco et al., 2009).

The embodied cognition view assumes that concepts arise directly from the perceptual experiences themselves. Take the example of the experience of dogs. When we perceive and interact with dogs, this leads to extraction of perceptual and functional attributes of dogs, which are stored in memory in *analog* fashion: our concept for ‘dog,’ on this view, closely resembles our perception and experience of a dog. When we imagine a dog, this is made possible by reactivating, or to use the technical term, *simulating* the perceptual and interoceptive experience of interacting with a dog—these include sensorimotor experiences when we pat and otherwise interact with a dog, as well as affective states, such as the pleasure we experience when a dog responds by wagging its tail, and so forth. But while the simulated dog closely resembles our conscious perceptual and interoceptive experience, it is, according to embodyists, attenuated.

In other words, the concept for ‘dog’ is not the same as the vivid experience of perceiving a dog. When we close our eyes and imagine a dog, we are at liberty to simulate an individual dog—perhaps our own pet—or a type of dog, or a dog composed of aspects of our past experiences of and with dogs. But the simulation is attenuated with respect to the perceptual experience of a dog—it doesn’t have the same vivid richness that comes with directly perceiving a dog in the flesh.

Importantly, the claim made by the embodied cognition perspective is that the simulation is directly grounded in the same brain states—in fact, a reactivation of aspects of the brain states—that are active when we perceive and interact with the dog. The simulation is then available for language and thought processes. As the reactivation of some aspects of the perceptual and interoceptive experiences of a dog is, in part, constitutive of the concept for ‘dog,’ the concept is an analog of the perceptual experience. It is analog in the sense that it is very much like our perceptual experience of dogs: the concept must, in part, be constituted of body-based representations—the sensorimotor experiences that comprise our perceptual experience—and, therefore, must be stored in the broadly the same brain regions that process the perceptual experience to begin with. This constitutes an embodied perspective as concepts are made-up, in part, of the very same body-based experiences that comprise our perceptual and interoceptive experiences.

Two main lines of empirical evidence suggest that the embodied cognition view of concepts, rather than the disembodied account, is on the right track. These relate to how the brain processes concepts, and how human subjects perform in behavioral tasks, when they must call up conceptual representations. Together, these two lines of evidence strongly suggest that concepts make use of the same brain regions that process the perceptual experiences that the concepts are representations of: it doesn’t matter whether you are perceiving a particular experience (percept), or later, thinking about it after the event (concept), the same brain states are activated in both cases. This suggests that the same mental substrate that underpins perception also underpins cognition, and our representations (or concepts) of perceptual experiences.

Brain-based demonstrations reveal that the brain’s sensorimotor and other modal systems—systems that are activated when we perceive a particular experience—are also activated during conceptual processing—when we think about or recall the experience, or even when we use or understand language relating to the experience. As we shall below, for instance, motor regions of the brain that are deployed for perceiving a particular tool, such as a hammer, and the way it is used, are automatically activated during non-perceptual tasks, such as thinking or talking about hammering. In short, a raft of studies provides clear evidence that the same motor processes in the brain are automatically engaged when subjects perform perceptual *and* conceptual tasks (Barsalou, 2003, 2008; Rizzolatti and Craighero, 2004; Gallese and Lakoff, 2005; Pulvermüller et al., 2005; Boulenger et al., 2008).

Behavioral demonstrations involve applying a stimulus of some kind to human subjects, and then observing their behavior when performing a particular task. Many of the relevant studies have involved sentence comprehension and lexical decision tasks (and I will have more to say about the relationship between language and concepts below).

However, one representative and important study required subjects to perform a lexical decision task employing action verbs relating to either arm or leg actions (Pulvermüller et al., 2005). The experiment made use of a technique, in cognitive neuroscience, known as transcranial magnetic stimulation (TMS). This is a non-invasive technique that involves passing a weak electric current, using electrodes attached to the scalp, to specific brain regions in order to stimulate them.

Subjects were asked to read words that related either to arm movement, such as *punch*, or leg movement, like *kick*. Immediately after reading, the TMS pulse was passed through either the leg region of the brain’s motor cortex or the arm region. Subjects were then asked to signal when they had understood the word. The experimenters found that when subjects received a pulse to the ‘arm’ region of the brain, they processed arm words more quickly. And when exposed to an electric current to the leg region, they understood leg words more quickly. What this reveals is that words—which relate to mental representations, concepts—were influenced by activation of the perceptual areas of the brain dedicated to perceiving either leg or arm actions. And consequently, this provides powerful evidence that perceptual experiences underpin conceptual representations, as manifested in language.

### The Nature of Simulations

If the linguistic and conceptual systems together constitute a meaning-making complex, how do simulations arise? A linguistically mediated simulation is a general purpose computation, performed by the brain, which provides language users with an approximation of a speaker’s linguistically mediated communicative intention.

The proposal is that words and other linguistic symbols are in fact cues that guide the way in which body-based states processed and stored by the brain are composed, in order to facilitate linguistically mediated meaning construction (Glenberg and Robertson, 1999; Fischer and Zwaan, 2008; Evans, 2009, 2013; Bergen, 2012: chapter 5). To illustrate, consider the use of *red* in the following example sentences:

(1a) The actress put on her red lipstick.
(1b) The red fox jumped over the stream.

In the first example, the use of *red* evokes a bright, vivid red. In the second, a dun or browny red is typically called to mind. This reveals that the meaning, or, more precisely, the perceptual simulation of *red*, is not, in any sense, there in the word. After all, *red* could, in principle, lead to activation of the full panoply of distinct hues we normally associate with red. These range, for instance, from the orange-red of fire, to the auburn-red of henna, to the crimson-red of blood, to the truly red of lipstick, and so forth. Knowledge of all these different shades arises from our interaction in and with the world, which we can, in principle, call to mind, and visualize in our mind’s eye in the absence of language.

In these sentences, the word *red* provides access to this meaning potential: all our stored experiences for red. But while the sensory experience of redness is not coming from language itself, the word cues the perceptual and interoceptive states stored in the brain, associated with red in all its glory. And these body-based states are reactivated during language use. Put another way, the word form *red* gives rise to distinct simulations for different hues of red.

But importantly, what’s remarkable about the meaning-making complex—the linguistic and conceptual systems-assembly—is that the sentences in (1) enable us to construct just the right shade of red: a contextually appropriate shade. The linguistic context, in each sentence, guides the construction of the simulation, such that we obtain the ‘correct’ perceptual hue in each case.

#### The Bifurcation in Semantic Representation Design Feature

If the function of language is to index or activate body-based concepts in the conceptual system, what is the difference between representations in the conceptual system vis-à-vis those in the linguistic system? The first design feature of linguistically mediated meaning construction, I argue in this section, constitutes a qualitative distinction in the two types of representation: *the design feature for a bifurcation in semantic representation*. This distinction I operationalise in terms of *analog knowledge* (indigenous to the conceptual system), and *parametric knowledge* (indigenous to the linguistic system).

### Arguments for Semantic Representations Indigenous to Language

There are a number of reasons for thinking that language comes equipped with semantic representations that are distinct from those that reside in the conceptual system—embodied concepts. I briefly review five here, based on Evans (2015a).

First, if language had no indigenous semantic content, we would be unable to use language to evoke ideas we haven’t yet experienced. This follows as the brain states wouldn’t yet exist for the corresponding experiences. But, it appears to be the case that language can do just that, facilitating the evocation of just those experiences not yet witnessed (Taylor and Zwaan, 2009; Vigliocco et al., 2009). For instance, I can describe a dance move to someone, using language, and more or less convey the move, even though my interlocutor may have never had previous experience of the move. While seeing and acting provide a directly perceived, multimodal context, enabling the formation of conceptual representations, an approximation can nevertheless be facilitated via language. While direct experience of the dance move—the experience of seeing, acting, and interacting, gives rise to body-based representations that are analog in nature—language, in contrast, doesn’t work like that. The representations are more sketchy. Nevertheless, language can be used, even in the absence of prior experience, in order to evoke a partial representation of the dance move. This demonstrates that conceptualisations can arise via the medium of language.

Second, although activations of body-based brain states arise automatically in response to language use, they are not necessary for language to be successfully used. Patients with Parkinson’s and motor neuron disease display difficulty in carrying out motor movements, as motor representations in the brain are damaged. Yet, both sets of patients are able to use and understand corresponding action verbs (Bak et al., 2001; Boulenger et al., 2008). This reveals that simulations arise not just from embodied brain states.

Third, language itself appears to encode a type of semantic representation that is qualitatively distinct from the sorts of rich, multimodal representations that populate the conceptual system. Consider, for instance, the semantic divergence between the use of the definite article, *the*, with the indefinite, *a*. One key distinction concerns specificity, as well as whether the information being introduced is already present or not, in the current discourse: whether the subject under discussion is given or new. That said, *the* and *a* don’t have specific referents in the world, nor are they ideas that can be visualized, in the way that, say the noun *dog*, or even a scene associated with the more abstract nominal *jealousy* can be visualized.

What this reveals is that so-called grammatical or function words appear to provide a relatively schematic semantic representation: a type of content that is qualitatively distinct from concepts. The grammatical structure of language may provide an indigenous level of semantic representation, distinct from non-linguistic concepts.

Fourth, language appears to directly influence perception. In one study, the distinction in the linguistic encoding of color was exploited to investigate the non-linguistic effects of language (Thierry et al., 2009). It was found that differences across languages, for instance, Greek vs. English, in terms of encoding of monolexemic color terms led to distinctions in the perception and categorisation of color space. This finding strongly suggests that language provides semantic content independent of the conceptual system, consequently leading to the cognitive restructuring in non-linguistic cognition.

The fifth reason relates to what I have termed, in earlier work, the illusion of *semantic unity* (Evans, 2009). Otherwise distinct aspects of semantic space can, under the influence of language, come to be viewed as unified. For instance, the polysemy exhibited by language can relate a number of distinct semantic parameters, providing the appearance of homogeneity. Take the English lexical item *over*, as in the following examples:

**Table:** 

(2a) The lamp is over the table	‘above’
(2b) The ball landed over the wall	‘on the other side’
(2c) The clouds are over the sun	‘covering/occluding’
(2d) The relationship is over	‘completion’

What these examples reveal is that in English a variety of distinct semantic parameters—‘above,’ ‘on the other side,’ ‘covering,’ and ‘completion’—are encoded by the same form. While the relationship between these semantic units is motivated (Tyler and Evans, 2003; Evans, 2015b), the units are nevertheless distinct. But the consequence of English employing the same form to encode a range of distinct—albeit semantically related—meanings, is that English speakers perceive the semantic units to form a coherent semantic range. In contrast, other languages divide similar semantic space across different lexical items. The consequence is that the appearance of semantic unity is just that, an illusion, an artifact of the way in which individual languages cut up and/or unify semantic space. It also provides further evidence that language provides a level of semantic content independent of the conceptual system, which it nuances during the process of meaning construction.

### Parametric vs. Analog Concepts

This discussion of the semantic content, derived via the linguistic system, and distinct from non-linguistic concepts, brings us to the design feature of linguistically mediated meaning construction under discussion in this section. The semantic content associated with a mental simulation appears to arise from a symbiotic coupling of two qualitatively distinct knowledge types. For instance, the content associated with so-called content words, such as the open-class noun *waiter*, self-evidently, relate to information “above” the level of language. When we imagine a waiter, this involves rich information concerning the appearance, dress, location, and tasks involved in being a waiter. Information of this kind is multimodal in nature, involving information that is sensorimotor and/or interoceptive. In short, it is *analog*—the information called to mind approximates the veridical “immersed” experience of perceiving and interacting with a waiter (cf. Zwaan, 2004).

In contrast, the so-called *function* or *grammatical* words and constructions concern information that is neither rich, nor multimodal, in the same way. In fact, the information conveyed is far more *schematic* in nature (Talmy, 2000; Evans and Green, 2006; Evans, 2009, 2013; see also Bergen, 2012: Chapter 5). To illustrate, if we exclude the semantic content associated with the open-class content words, in (3), we are left with a type of schematic representation that is not straightforwardly imageable, or perceptual. In short, the representations associated with grammatical structure, appear not to relate, in a straightforward way, with perceptual representations. And yet, such representations are nevertheless meaningful:

(3) **Those** decorator**s are** ruin**ing my** wall**s**

In (3), by excluding the content words—*decorator, ruin* and *wall*—what remains is the function words, which I’ve highlighted in bold font. These are the inflections *–ing* and *–s* and the lexical items *those, are*, and *my*. In addition, the grammatical categories noun and verb also encode schematic semantic units, those of THING and PROCESS, independently of the specific lexical items that fill them—*decorator, wall* and *ruin* (Langacker, 1987, 2008; Evans, 2015b). So, the semantic representation of just these closed-class elements, together with the syntactic configuration in which they are embedded, can be captured as in (4):

(4) *Those somethings are somethinging my somethings*.

The gloss for this semantic representation can be provided as in (5):

(5) More than one entity close to the speaker is presently in the process of doing something to more than one entity belonging to the speaker. This provides quite a lot of semantic content.

That said, this semantic representation is, nevertheless, highly schematic. We don’t have the details of the scene: we don’t know what the entities in question are, nor do we know what is being done by the agent to the patient. Nevertheless, this illustration reveals the following: there appears to be a type of semantic representation that is unique to the linguistic system. Moreover, this representation provides information relating to how a simulation should be constructed (see Bergen, 2012 for a related point).

After all, the grammatical organization of the sentence entails that the first entity is the agent and the second entity the patient: the first entity is performing an action that affects the second entity. This level of semantic representation derives exclusively from language, rather than from representations in the conceptual system. It provides a set of instructions as to the relative significance, and the relation that holds, between the two entities in the sentence. In short, the function words and the grammatical construction—in the sense of Goldberg (1995, 2006)—involves semantic content, albeit of a highly schematic sort (Talmy, 2000; Evans, 2009).

This distinction, in terms of the nature of the content associated with content words on the one hand, and function elements on the other, constitutes the second design feature for human meaning construction. Words like *decorator, ruin* and *wall* give rise to rich experiences, which are analog in nature: they relate to entities which we have directly experienced and about which we retain detailed knowledge. Accordingly, I refer to knowledge of that sort as *analog concepts*—concepts that are directly grounded in the experiences that give rise to them. How then does semantic structure (in language) differ from this level of conceptual structure—which is to say, from analog concepts?

To illustrate, consider the use of the adjective *red*, and the noun *redness*:

(6a) The bee sting caused a **red** mark on her hand.
(6b) The bee sting caused **redness** on her hand.

In both instances, the same perceptual hue is evoked, caused by the toxin we attribute to bee stings. But the simulation associated with the sentences is slightly distinct. In the first example, *red*, an adjective, gives rise to an interpretation in which the person’s hand has the property of being red, a consequence of the bee sting. But in the second, the bee sting causes a particular ailment, deriving from the use of the noun redness.

As we have seen, a noun encodes a semantic unit: THING; this is what I refer to as a semantic parameter—a schematic semantic ‘atom’ of meaning, one specialized for being encoded in language (Evans, 2015b,c). In contrast, an adjective encodes the parameter PROPERTY (OF A THING). The consequence of the grammatical categories noun vs. adjective encoding distinct parameters is that the way in which the conceptual structure—the mental representation of red that resides in the conceptual system—becomes activated is nuanced by the language-specific representations—the parameters—encoded by grammatical structure. In short, the interpretation deriving from each of the examples in (6) diverges in subtle, albeit important ways. The interpretation arising from (6a), that the perceptual hue arises due to a skin property, is due to the use of the adjective. In contrast, the interpretation in (6b), with a divergent simulation, that of a skin ailment, is a consequence of the use of the noun. Put another way, language provides a level of knowledge that is more schematic—I use the term parametric—than the rich, analog concepts—available from the conceptual system. And these semantic parameters, specific to language, I term parametric concepts.

My proposal is that analog concepts—which are semantic representations that populate the conceptual system—in evolutionary terms, had to precede the existence of language. Parametric concepts constitute a species of concept that arose as a consequence of the emergence of language. They provide a level of schematic representation directly encoded by language: parametric concepts guide *how* analog concepts are activated and, consequently, *how* simulations are constructed in the service of linguistically mediated meaning construction. For instance, the forms *red* and *redness* both index the same perceptual state(s). But they package the conceptual content in a different way, giving rise to distinct simulations: *redness* = ailment; *red* = property of skin. The schematic parametric concepts, which is to say, that part of semantic representation that is native to language, relates to THING vs. PROPERTY. Parametric concepts are *language-specific affordances*, rather than affordances of the conceptual system.

## The Bifurcation in Linguistic Organization Design Feature

While I’ve presented a proposal that there is a distinction between two semantic representational systems—the conceptual vs. the linguistic, which are each populated by qualitatively distinct representations—analog vs. parametric—the second design feature for linguistically mediated meaning construction relates to language itself. Language exhibits a bifurcation in terms of the nature of the linguistic symbols that populate it: *the design feature of a bifurcation in linguistic organization*. And this design feature is fundamental in terms of the enabling language to engage with the representations in the conceptual system, and hence, in terms of guiding the parcellation of analog knowledge in meaning construction.

### Two Types of Symbolic Reference

**FIGURE 1:**
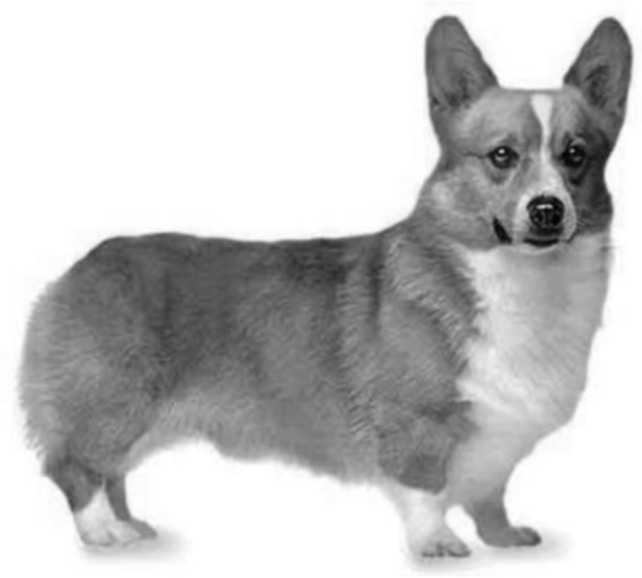
**A dog**.

Language appears to employ two qualitatively distinct types of symbolic reference (Evans, 2015b). The first constitutes what I dub a *words-to-world direction* of symbolic reference: the type of symbolic reference which de Saussure (1916) largely focused on. In this type, signs are conventionally associated with specific objects and events in the world, and/or in the mind of the language user. The symbolic relation holds between a referential vehicle from the linguistic system, and an entity or idea outside the system. For instance, the English word /d6g/ refers to the pet of choice for many western households, as represented in **Figure 1**.

**Figure 1** captures this type of relation. It shows that a given sign—sign1, sign2, and so forth—is symbolically related to objects and events in the world and/or the mind. The symbolic relation, established by convention, is represented by the directed arrow, connecting a particular sign with its referential target.

Importantly, the nature of the referential target constitutes a potentially large body of knowledge that you and I may have concerning dogs, knowledge which is dynamically updated: each time you step outside your front door, and see a dog across the street, your knowledge is updated, and the symbol refers not just to specific exemplars of dogs, for instance, a Welsh Corgi, depicted in **Figure 1**, but other breeds too. It may also include a wide range of knowledge you possess concerning dogs, including their behavior and life cycle, their appearance, their status in human life and culture, as well as a plethora of information you’ll have gleaned through direct experience with dogs, including dogs you may have known, as well as information derived through cultural transmission. Hence, the referential target of a sign in fact relates to a complex web of knowledge, what I term the *semantic potential* of the target—developed in my theory of *Access Semantics* (Evans, 2009, 2013, 2015b).

**FIGURE 2:**
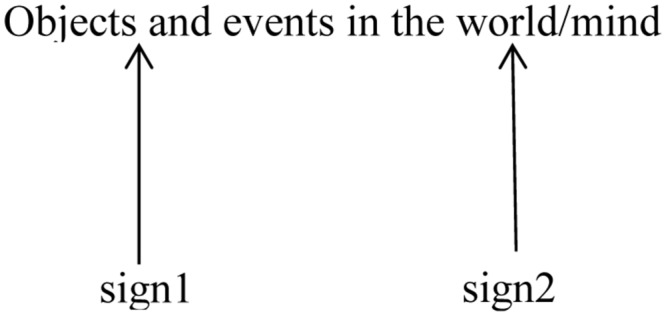
**Words-to-world symbolic reference**.

The second symbolic reference strategy involves what I dub a *words-to-words direction* of symbolic reference (**Figure 2**). Here, the symbolic relation holds not between a sign, and an entity in the world and/or the mind. Instead, reference holds between one linguistic symbol and another. To illustrate, consider the following referring expression:

(7) a dog

While the noun phrase (NP), *a dog*, as a whole, refers in a words-to-world direction, the indefinite article refers to the noun (N), *dog*: it has a words-to-words direction of reference. Indeed, the *semantic function* of the indefinite article is specialized for words-to-word reference: whatever it is that the symbol, *dog*, refers to, the indefinite article tells us that the sign to which it refers, in this case, *dog*, is both univalent—there’s just one of it—and non-specific—the hearer can’t be expected to have specific information about the entity; it is for this reason that the symbol *a* is termed the ‘indefinite’ article.

One way of thinking about the indefinite article is that, in part, it encodes a schematic slot—what has been termed an *elaboration site* (Langacker, 1987) –which is completed by a noun. In short, the English indefinite article requires a noun to elaborate it, and hence to complete its meaning. Notice that while the overall function of the referring expression—*a dog*—is to identity an individual entity in the world—a words-to-world direction of reference—the English symbol *a* is specialized for a words-to-words direction: it assumes a distinction in *lexical classes*, such as noun vs. indefinite article.

Now consider a more complex example of words-to-words symbolic reference, focusing on the noun *aim*, in the following attested example:

(8) The Government’s **aim** is to make GPs more financially accountable, in charge of their own budgets, as well as to extend the choice of the patient. (Schmid, 2000).

In (8), *aim* can be thought of as a *shell noun* (Schmid, 2000)—it refers to the entire conceptual complex that I’ve underlined. The underlined portion of the discourse chunk, whilst, on the face of it, relating to a complex set of ideas, is encapsulated as a coherent conceptual whole. Importantly, this is achieved via word-to-word symbolic reference: the noun, *aim*, provides a linguistic “shell,” enabling reference to the complex idea that it points to. Evidence for this function comes from the next sentence in the discourse, which I present below:

(9) The Government’s **aim** is to make GPs more financially accountable, in charge of their own budgets, as well as to extend the choice of the patient. Under **this new scheme**, family doctors are required to produce annual reports for their patients…(Schmid, 2000).

Having established a *shell noun complex*—the underlined portion—by virtue of a referring shell noun, *aim*, it is then possible to continue treating the complex as a single coherent conceptual entity, in ongoing discourse. Evidence for this comes from the new shell NP, *this new scheme*, which, again, I’ve highlighted in (9). This shell NP refers back to the underlined shell noun complex, established by the symbol *aim*, in the first sentence of the discourse chunk. In short, both *aim* and *this new scheme* refer symbolically in a words-to-words fashion, providing a means of packaging a complex idea—a shorthand mnemonic—without the need to continue to spell out the entire idea itself.

**FIGURE 3:**
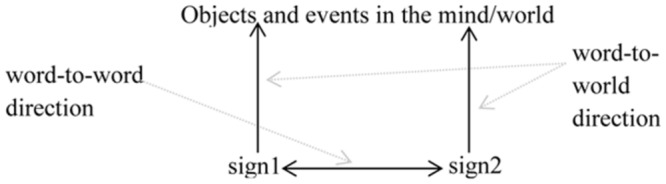
**Two types of symbolic reference**.

Language, then, appears to make use both of words-to-world and words-to-words types of symbolic reference. **Figure 3** captures both directions of symbolic reference.

These two types of symbolic reference, exploited by language, are qualitatively different: words-to-words symbolic reference is more abstract than words-to-world symbolic reference. This follows as reference in this direction is to another symbol, rather than to an idea or entity in the world (or mind) *per se*. It presumes the existence of a linguistic level of semantic representation which can be referred to, independently of entities in the world. Moreover, this distinction reflects a fundamental design feature of language: the distinction between a *lexical system* and a *grammatical system*.

### How do the Two Reference Strategies Contribute to Meaning Construction?

The words-to-world referential strategy constitutes our ability to use linguistic symbols to cue or activate representations in the conceptual system. In contrast, the words-to-words strategy constitutes the means, afforded by the linguistic system, to construct the *semantic scaffolding* for a simulation. The semantic scaffolding enables the relevant part of the conceptual system’s vast semantic potential to become activated during meaning construction. In short, words-to-words reference provides the basis for words-to-world reference to narrow in on just that aspect of the conceptual system that is relevant for linguistically situated meaning construction, as when the word *red*, in (1) enabled activation of two distinct perceptual hues. To illustrate the way in which this works, consider the following linguistic example:

(10) A waiter served the customers.^1^

This sentence features words, and other linguistic constructions, that serve two distinct reference strategies. Let’s consider the words-to-world strategy first. This strategy equates, in linguistic terms with the so-called *content words* in the sentence. I’ve highlighted these in bold in (15):

(11) A **waiter serve**d the **customer**s.

A content word, as discussed earlier, usually taken to be a word that concerns rich content. In (11) these are the nouns *waiter* and *customer*, and the verb *serve*. These words relate to relatively rich aspects of the scene being described, in particular, the participants in the scene and the relationship that holds between them. Moreover, we are able to map these words onto rich and detailed scenarios, stored in the conceptual system, relating to our experience of interacting in the world. We each have rich, and varied experiences of restaurants, eateries and other venues that sell food for consumption *in situ*, including the format and moves involved in such service encounters. We know that a waiter is someone who liaises with the customer on choice of food, and the kitchen where the food is prepared. The waiter’s function is to communicate with both parties, and to deliver the food, once prepared, to be consumed by the customer, in return for pay, and often, for a tip. In short, these content words encode a words-to-world relation: they enable language users to map the words onto specific participants and the relations holding between them; in slightly different terms, they facilitate to the rich analog knowledge that resides in the conceptual system: knowledge we have about a restaurant *frame*.

In contrast, the sentence also consists of function words and grammatical constructions (within which the content and function elements are embedded). I’ve placed the function elements in bold in (12):

(12) **A** waiter serve**d the** customer**s**.

Function words encompass those schematic notions which, in the most simplistic of terms, aren’t imageable. For instance, while we can call to mind, should we wish, a waiter or a customer, or imagine what is entailed by a waiter serving a customer, it’s not clear what is called to mind by grammatical words such as *a*, or *the*, the past tense marker –*ed*, or the bound plural morpheme –*s*. These elements, on their own, are specialized not for indexing particular entities in the world *per se*. Rather, their function is to say something about how we should interpret the other words in the sentence that they relate to. For instance, the past tense marker constrains our interpretation of the verb *serve*: it situates the serving event as having taken place before now. But in this way, the past tense marker is guiding the way in which, whatever it is in our conceptual system that *serve* facilitates access to, the way this knowledge becomes activated. Similarly, the plural marker provides a means of interpreting the free morpheme, the noun *customer*, to which it is morphologically bound.

One line of evidence for distinguishing between content and function words, between words-to-world and words-to-words reference, takes the following form: if we change the content words, we obtain a different scene, yet the structural elements, provided by words-to-words reference, remain the same. Consider the following:

(13) A rockstar smashed the guitars.

In (13), when changing just the three content words an entirely different experiential complex—a simulation—arises, one involving a rockstar smashing guitars. This reveals that the function of words-to-world reference concerns people, things, events, properties of things and events, and so on. But the semantic scaffolding remains the same, as the words-to-words relations are unchanged: *a, -d, the* and*-s*. These aspects of the sentence concerns whether the participants (rockstar/guitars) evoked can be easily identified by the interlocutor (the use of the indefinite article *a* vs. the definite article *the*), that the event took place before now (the used of the past tense marker, *-d*), and how many participants were involved (the presence, or absence, of the plural marker *–s*).

Moreover, the semantic scaffolding provided by words-to-words reference encompasses more than just the function words. It also includes the full range of grammatical constructions in which the content words participate. This includes the lexical class in which words participate: *waiter* and *customer* are nouns, while *serve* is a verb, as well as word order—in these example sentences, we have a declarative word order. And finally, the sentences all invoke active, rather than passive voice. In each case, these grammatical constructions—lexical class, word order, and voice—all facilitate a words-to-words referential strategy: they constrain how we should interpret the participants in the event, and the nature of the relationship holding between them.

Let’s focus on lexical class first. Consider the following expressions:

(14a) thumb a lift
(14b) lift a thumb

While the expressions in (14) involve the same phonological forms, *lift* and *thumb* belong to different lexical classes in each expression. In (14a) *thumb* is a verb, and *lift* is a noun. In contrast, in (14b) *lift* is a verb and *thumb* a noun. This follows because, one of the things we happen to know about English is that the article typically precedes a noun. And on the basis of this distributional analysis, *lift*, in (14a) and *thumb* in (14b) are nouns. Moreover, because we also know that verbs can serve an imperative function, especially when they appear in first position in an expression, *thumb* is a verb in (14a), while *lift* is a verb in (14b).

Now, the fact that the same phonological forms can shift their lexical class, as they do in these examples, reveals that the lexical classes, noun vs. verb, is a functional category independent of the phonological forms themselves. The categories noun vs. verb have functional significance independently of their lexical instantiations, and serve to constrain how we should interpret the phonological forms, and their referential targets, in each case. In (18a), consequently, the scene involves a hitch-hiking scenario, whilst in (14b) a different scenario is evoked, involving physical movement of someone’s anatomy.

Similarly, the declarative word order in (10) signals that the scenario being evoked is one that the speaker knows, or assumes to be true, and is presenting it as such to the interlocutor. If we alter the word order, by adding the function word *did* so that waiter is no longer the first element in the sentence, as in (15), we no longer have a declarative construction, but rather an interrogative. And now we have a different perspective on the scenario: the speaker is no longer presenting the scenario as fact, but, in fact, signaling that they don’t know whether the scenario is true.

(15) Did a waiter serve the customers?

What this shows is that the declarative, and indeed, interrogative word orders, in English constrain in rather important ways the way the information—the words-to-world strategy— is being packaged. Moreover, the ideational function and hence interactive-interpersonal function of both sentences is rather different: (15) invites a response in a way that (10) doesn’t.

And finally, active voice designates a particular point of view, which constrains the nature of the relationship holding between the participants in a scene. In (10), the point of view is being designated as located with the agent—the waiter. If we change the grammatical construction to passive, as in (16), the point of view is now situated with the customers, even though the waiter remains the active participant—the agent—in the words-to-world relation designated:

(16) The customers were served by a waiter.

The upshot of all this is that while the content of the simulation is achieved by language working to provide a structure for analog concepts—the scaffolding upon which the scene is constructed—language both affects, and consequently transforms, in significant ways non-linguistic content; in short, the conceptual content is packaged, for communicative purposes in the course of linguistically mediated meaning construction, by virtue of language-specific representations. **Table 1** provides a summary of what is conveyed by function words for sentence (14), whilst **Table 2** provides a summary from the perspective of analog concepts, *accessed* by content words (see Evans, 2009).

**Table 1:** Content deriving from words-to-world referring expressions.

Phonological vehicle	Words-to-world relation
Waiter	Person with a particular function, and sometimes appearance, who works in a particular setting
Serve	Particular mode of activity involving two or more people and, typically, an entity with which one of the participants is provided by the other
Customer	Person who is provided with a particular object or service (of various sorts) in exchange for, typically, money

**Table 2:** Content deriving from words-to-words referring expressions.

Phonological vehicle	Words-to-word relation
A	Introduces a referent which the hearer is held to be unable to readily identify (from context or preceding discourse)
A	Designates a unitary instantiation of the referent
The	Introduces a referent which the hearer is held to be able to readily identify (from context or preceding discourse)
–s	Designates multiple instantiations of a referent
Lexical class: verb (for *serve*)	Designates entity as an event (as one possibility)
Lexical class: noun (for *waiter/customer*)	Designates entity as an object (as one possibility)
Grammatical relation: subject (for *waiter*)	Designates entity as being the primary or focal entity in a designated relationship
Grammatical relation: object (for *customers*)	Designates entity as less important or secondary entity in a designated relationship
Active voice (through verb form)	Designates point of view being situated at the agent
Declarative word order	Speaker knows the situation to be true and asserts it to the hearer

### Linguistic Access to the Conceptual Meaning Potential

The lexical vs. grammatical subsystems can be analyzed in terms of words-to-world and words-to-words alignment, and in terms of analog vs. parametric knowledge. And this provides the critical design feature for meaning construction.

I have argued that analog knowledge does not in fact coming from language: the distinction between lexicon and grammar provides a design feature for access to the conceptual system—open-class words provide access, while the grammatical system facilitates the parcellation of analog knowledge to which open class words facilitate access. In short, a class of lexical elements, which I have loosely referred to as content words, and in English, associated most notably, but perhaps not exclusively with the ‘big four’—noun, verb, adjective and adverb—facilitate access to analog knowledge, to the conceptual system. On this Access Semantics account (aka The Theory of Lexical Concepts and Cognitive models, or LCCM Theory, developed in Evans, 2009, 2013, 2015a), language has a ready-made means of facilitating access to a type of knowledge not present within the linguistic system. It can reuse existing knowledge, evolved for other means than communication, for purposes of communication. The words-to-words function of the grammatical subsystem enables the parcellation of analog knowledge and hence a means of sophisticated meaning construction. As we’ve seen, knowledge of this type is schematic, providing a semantic scaffolding that nuances the analog information, giving rise to complex and subtle meaning, as in the distinction between a skin condition rather than unwanted colouration of the skin, as in the examples in (6).

On this account, a subset of linguistic symbols provide access to the conceptual system: in both words-to-world, and words-to-words directions; *red* and *redness* provide both types of symbolic reference. The parcellation of knowledge associated with analog information is driven by the parametric content conventionally associated with these forms: whether the perceptual hue is interpreted as a property of an entity or an entity in its own right, reified independently of whatever it happens to be a property of.

## Conclusion

In this paper, I have examined proposals for two central design features of the human capacity for linguistically mediated meaning construction: a bifurcation in semantic representation, and a bifurcation in linguistic organization. The striking claim to emerge is that language is tightly coupled with non-linguistic representations, in the conceptual system, which evolved not for communication. But language has evolved in order to bootstrap these representations for linguistically mediated communication.

The over-arching design feature of the human meaning-making capacity amounts to two distinct representational systems: the conceptual system and the linguistic system. Each system contributes to meaning construction in qualitatively distinct ways. The second is, given that the two systems are representational—they are populated by semantic representations—the nature and function of the representations are qualitatively different. After all, as a linguistic system has a different function, vis-à-vis the conceptual system, which is of far greater evolutionary antiquity, then the semantic representations are complementary, and as such, qualitatively different, reflecting the functional distinctions of the two systems, in collectively giving rise to meaning.

And finally, language itself is adapted to the conceptual system—the semantic potential—that it marshals in the meaning construction process. Hence, a linguistic system itself exhibits a bifurcation, in terms of the symbolic resources at its disposal. This relates to two distinct reference strategies available to linguistic symbols: words-to-world reference and words-to-words reference. In slightly different terms, this design feature of language amounts to a distinction between a lexical subsystem, and a grammatical subsystem.

The overall conclusion to emerge from this discussion is the following. The ideational function of language—its communicative potential—is, in large measure, a function of the way in which it is adapted to, and interfaces with the conceptual system. Rather than language being a distinct module or faculty of mind, it subserves meaning construction through a close and symbiotic relationship with the conceptual system: it has evolved and is designed to exploit those non-linguistic representations for purposes of linguistically mediated communication. But to achieve this, it has evolved a means of words-to-words symbolic reference—a grammatical capacity—which appears to be a species-specific trait. And it is the parametric knowledge units, associated with morphosyntax and lexical items, that enables our species to harness the otherwise mute semantic potential of concepts in order to convey meaning.

## Author Contributions

The author confirms being the sole contributor of this work and approved it for publication.

## Conflict of Interest Statement

The author declares that the research was conducted in the absence of any commercial or financial relationships that could be construed as a potential conflict of interest.

## Footnotes

1 Example based on Evans (2009, p. 102).
